# The perilous path: reimagining promotion and tenure in African academia

**DOI:** 10.11604/pamj.2024.49.5.44855

**Published:** 2024-09-03

**Authors:** Alphonsus Neba, Luchuo Engelbert Bain

**Affiliations:** 1 African Population and Health Research Center (APHRC), Nairobi, Kenya

**Keywords:** Tenure, merit, academia, Africa

## Abstract

The current scientific publication architecture and business models are structured to privilege high-impact Western journals. This has been perpetuated in academia and by funding agencies, where a researcher´s value is often measured by the number of publications and where these papers are published. However, the current system renders journals from low- and middle-income countries, including African journals, largely invisible. Indeed, it is important to answer the fundamental question of why we conduct research. Most would argue that research is only ethically grounded if its core purpose is to create impact and improve lives. There is compelling evidence that the time lag from evidence generation to translation into policy is about 17 years. There is no evidence that publishing in a high-impact journal is more likely to create an impact. Indeed, the move by many universities away from using the impact factor as a measure of academic value is increasingly welcomed. It has become critical to redefine and restructure academic merit. In this essay, we will explore the Afro-centric dimensions of the publish-or-perish rhetoric and its impact on tenure in African academia. We argue that context-relevant and alternative metrics are needed to redefine academic merit, as well as the intentionality of African governments and universities to invest in, trust, and value their own journals as non-negotiables in giving African journals the visibility and trust they deserve. We present an African Population and Health Research Centre (APHRC) approach, supported by the Gates Foundation, intentional in decolonizing the global publication space, and clearly aligns with its mission of improving lives in Africa through research.

## Essay

The air hangs heavy with anticipation in the cramped faculty common room. Dr. Abena, a rising star in Ghanaian immunology, clutches the freshly printed copy of her research. Months of meticulous lab work, fueled by late nights and unwavering determination, culminate in this moment of triumph. Her findings, a novel approach to tackling a prevalent childhood disease, holds immense promise for improving public health in Africa. Yet, a shadow of doubt creeps in as she contemplates the next step: publication.

**The gauntlet of "publish or perish": a stifling reality:** the air in the room crackles with unspoken anxieties. Dr. Abena glances around at her colleague Professor Kwame, a seasoned malaria researcher, hunched over his laptop, his brow wrinkled in frustration. Dr. Abena knows his struggle. He, too has a ground-breaking discovery, a potential breakthrough in malaria treatment. But the pressure to publish in high-impact, Western journals the unspoken currency of academic success weighs heavily on him. Tenure, the golden ticket to academic stability, seems an impossible dream without a coveted publication record. Professor Kwame, Dr. Abena's esteemed mentor, casts a knowing smile. Her weathered face, etched with the lines of countless battles fought, reflects a deep understanding of the treacherous path they navigate. "The gauntlet awaits, my dear," she says, her voice laced with a bittersweet mix of empathy and resignation. The gauntlet of “publish or perish." This pervasive mantra defines the current promotion and tenure (P and T) system in many African universities and research institutions. The system incentivizes a relentless pursuit of publications in prestigious Western journals, often neglecting the immense value of research addressing African challenges published in African journals. Dr. Abena's research, deeply relevant to the Ghanaian context, might struggle to find traction in these journals with their Western-centric focus. This creates a vicious cycle; valuable research remains unseen, further diminishing the visibility and impact of African journals.

Professor Kwame slams his laptop shut, the frustration evident on his face. "Another rejection," he sighs. "They want more 'novelty,' more 'generalizability.' As if malaria isn't a problem unique to Africa!" Dr. Abena nods sympathetically. The irony stings: research desperately needed in Africa often gets overlooked by journals chasing global relevance. The current P and T system, far from nurturing innovation for African solutions, stifles intellectual exploration and discourages research that could lead to ground-breaking solutions for African problems. This is the perilous path that Dr. Abena, Professor Kwame, and countless other brilliant African researchers must navigate. It's a path demanding a paradigm shift, a bold reimagining of P and T practices that fosters a vibrant, Africa-centered research ecosystem. This essay delves into the critical need for such a transformation. It proposes a multi-pronged approach that values the diverse contributions of African scholars, recognizes the vital role of African journals, and incentivizes research relevant to the continent's needs. It's a call to action for universities, research institutions, funding agencies, and policymakers to join forces and forge a path for a thriving research landscape in Africa, one that empowers ground-breaking research like Dr. Abena's and transforms the future of the continent.

**The mirage of visibility: the invisibility of African journals:** the current P and T system privileges publications in high-impact Western journals, often neglecting the crucial role African journals play [1]. These journals address critical issues impacting the continent, from neglected tropical diseases to the socio-economic consequences of climate change. Here, African researchers find a platform for research that speaks directly to their realities, influencing policy and development strategies. However, the current system renders African journals largely invisible. Many lack the resources to secure prestigious indexing, hindering their global reach. Citation rates, a key metric in P and T evaluations, often suffer due to a lack of awareness and ingrained biases towards research from the Global South. This creates a vicious cycle; valuable research remains unseen, further diminishing the visibility and impact of African journals.

**Beyond "publish or perish": redefining academic merit:** the tyranny of the "publish or perish" model stifles intellectual exploration and genuine innovation. Promotion and tenure evaluations urgently need to move beyond a largely favored singular focus on publication quantity and impact factors. A holistic approach that considers the quality, relevance, and societal impact of research is paramount. Here's where Dr. Abena's work shines. Her research, published in a reputable African journal, directly tackles a health concern plaguing her country. Its potential to improve public health outcomes is undeniable. Promotion and tenure committees must recognize such research as a valuable contribution, irrespective of the journal's Western pedigree.

**Beyond publications: recognizing the multifaceted contributions of African scholars:** African academics wear many hats. Teaching, mentoring, community engagement, and policy advocacy are all integral parts of their roles. Yet, these contributions often go unrecognized in P and T evaluations. We need a more nuanced system that acknowledges the diverse ways scholars contribute to the academic landscape. Dr. Abena, for instance, dedicates significant time to community outreach programs, educating parents about the disease she researches. Her passion for translating scientific knowledge into tangible benefits for her community deserves recognition alongside her publications. Emphasizing such contributions will create a more holistic picture of academic merit and encourage a well-rounded approach to research.

**Building a collaborative ecosystem: towards an Africa-centered approach:** the current P and T system operates in a siloed environment, neglecting the potential of collaboration. Imagine a network of African universities and research institutions collaborating on research agendas, jointly creating high-quality, peer-reviewed African journals with strong editorial boards. This would not only enhance the visibility of African research but also foster intellectual exchange and strengthen the research ecosystem across the continent. Universities can spearhead this change by establishing regional partnerships, creating interdisciplinary research centers, and facilitating knowledge-sharing platforms. Collaboration with international partners, when done on equal footing, can further enrich the research ecosystem.

**Transforming the narrative: decolonizing knowledge production:** the current P and T system reflects a legacy of colonial knowledge production [2]. It's crucial to recognize the inherent bias towards Western research methodologies and epistemologies. African researchers need the freedom to pursue research that draws on indigenous knowledge systems and methodologies relevant to their contexts. By valuing African perspectives and promoting research that addresses African challenges, we can start decolonizing knowledge production. Dr. Abena's work, deeply rooted in the African context, exemplifies the value of such research. African scholars have to be intentional in living the reality of intellectual confidence. Indeed, a change in mindset to value their own (knowledge and journals) is highly needed. Some countries (e.g. South Africa) are taking the lead in encouraging scientists to value and publish in local journals.

**Empowering the next generation: fostering a culture of innovation:** Dr. Abena's career trajectory represents the plight of many young, talented African researchers. The current P and T system discourages them from pursuing research relevant to Africa's needs, pushing them towards generic topics with a higher chance of publication in Western journals. This stifles innovation and discourages research that could lead to ground-breaking solutions for African problems. We need to cultivate a culture of intellectual risk-taking. Promotion and tenure evaluations should encourage researchers to explore unexplored territories, addressing critical issues specific to Africa. This requires a shift in mindset among senior academics, who hold significant sway in P and T committees. Mentorship programs that encourage young researchers to pursue impactful research within an African context are crucial. Dr. Abena needs mentors who understand the value of her work, not just chase the next high-impact publication.

**Incentivizing open access: sharing knowledge for societal impact:** the current model often prioritizes publications behind expensive paywalls, hindering knowledge dissemination and limiting the impact of research. Open access journals, freely available to all, can revolutionize how research is accessed and utilized. Promotion and tenure committees can incentivize the publication of research in open access African journals, ensuring wider dissemination of knowledge for maximum societal impact. Imagine the ripple effect if Dr. Abena's findings were readily accessible to other researchers and policymakers across Africa, accelerating the development of interventions for the disease she investigates [3].

**Redefining impact: beyond citation metrics:** the overreliance on citation metrics in P&T evaluations fails to capture the true impact of research, especially within the African context. Metrics that measure the practical application of research findings, such as its influence on policy changes, community engagement initiatives, or real-world interventions, need to be considered. Dr. Abena's research might not garner a high number of citations in the initial stages. But if her findings inform the development of a successful public health campaign or a new treatment strategy, its impact becomes undeniable. We need P and T committees that value such outcomes and incentivize research that leads to tangible benefits for African communities.

**Case studies of reimagining promotion and tenure in action**

**The University of Cape Town's (UCT) research impact framework:** the University of Cape Town (UCT) serves as a leading example of a university actively reimagining its P and T practices. In 2018, UCT adopted a research impact framework that moves beyond a sole focus on publications. The framework recognizes a diverse range of research outputs, including: i) Books and chapters published by African presses; ii) Research informing policy changes or public health interventions; iii) Community-engaged scholarship and partnerships; iv) Contributions to open educational resources. This holistic approach allows researchers to showcase the broader impact of their work, particularly research addressing local challenges published in African journals. The framework also emphasizes societal engagement activities, such as public lectures, media outreach, and community workshops. This incentivizes researchers to translate their findings into actionable knowledge that benefits the public.

**The African population and health research center (APHRC): building an enabling environment:** the African Population and Health Research Center (APHRC) serves as another noteworthy example. African population and health research center, a leading research institution in Africa, recognizes the importance of nurturing a supportive environment for early-career researchers. They offer mentorship programs that guide young researchers towards impactful research aligned with African priorities. Additionally, APHRC provides training workshops on research methods, scientific writing, and publication strategies, specifically tailored for African journals. This empowers researchers to navigate the publication landscape and contribute their findings to journals with high visibility within the African context. These case studies illustrate how universities and research institutions can implement innovative P and T practices that value and incentivize research relevant to Africa. By adopting a multi-pronged approach that recognizes diverse research outputs, promotes societal engagement, and fosters a supportive environment for early-career researchers, African institutions can create a fertile ground for impactful research that thrives within an Africa-centered ecosystem.

**Metrics for evaluation societal impact: beyond citation counts:** the current reliance on citation metrics in P and T evaluations fails to capture the true impact of research, particularly in the African context. Here are some alternative metrics that can be considered: i) Policy impact: track instances where research findings directly inform policy changes or the development of new government initiatives. This could involve analyzing policy documents, conducting interviews with policymakers, or tracing the influence of research on legislative debates; ii) Community engagement: measure the extent to which research involves and benefits the communities it investigates. This could involve quantifying the number of participants in research projects, tracking the implementation of community-based interventions, or assessing the level of community ownership over research outcomes. iii) Media coverage: analyze the extent to which research findings gain public attention and influence public discourse. This could involve tracking media mentions, analyzing the framing of research in news coverage, and assessing the impact of research on public awareness of key issues. iv) Mentorship and capacity building: consider the extent to which research contributes to the development of future generations of researchers. This could involve tracking the number of students mentored, the creation of new training programs, or the establishment of research collaborations with African universities. v) Downloads and online engagement: in the era of open access publishing, track the number of times research articles are downloaded, shared on social media, or used as reference materials in online courses. This provides insights into the reach and utilization of research findings beyond traditional academic circles. vi) Value and quality of local journals: reward reviews in local journals (African), and consider this a metric within the academic promotion package. Developing a robust framework for measuring societal impact requires careful consideration and adaptation to specific research contexts. However, the above metrics can serve as a starting point for moving beyond the limitations of citation counts and creating a more comprehensive picture of the true impact of research on African communities.

**The role of funding agencies: incentivizing Africa-centered research:** international and African funding agencies play a crucial role in shaping the research landscape in Africa. They can play a significant role in incentivizing research addressing African challenges and published in African journals by adopting the following strategies: i) Thematic funding calls: dedicate funding streams to research addressing specific African priorities, such as neglected tropical diseases, climate change adaptation, or food security. These calls should explicitly encourage publication in reputable African journals with strong editorial boards. ii) Open access publishing support: offer financial support to enable researchers to publish their findings in open access African journals. This could involve covering publication fees or establishing dedicated grant programs specifically for open access publishing initiatives. iii) Capacity building grants: allocate funding for capacity-building programs that equip African researchers with the skills necessary to conduct high-quality research and publish effectively in African journals. This could include training workshops on scientific writing, editorial processes in African journals, and research methodologies relevant to the African context. iv) Collaborative research partnerships: encourage and incentivize South-South and North-South research collaborations that focus on African challenges. Funding agencies can play a role in facilitating these collaborations and ensuring that research findings are disseminated and utilized within Africa. v) Evaluation criteria: revise evaluation criteria for research proposals to consider the potential societal impact of research within Africa. This could involve incorporating metrics like anticipated policy influence, community engagement strategies, and potential for capacity building within African research institutions. Furthermore, revise grant evaluation criteria to acknowledge the value of research published in African journals. vi) Reviewers should be trained to assess the quality and relevance of research within the African context, not solely based on the journal's impact factor or Western-centric benchmarks. v) Journal ranking reconsideration: move beyond a sole reliance on established journal ranking systems that often overlook the value of African journals. Funding agencies can advocate for the development of alternative ranking systems that recognize the quality and relevance of African journals within their specific contexts. International and African funding agencies have a significant responsibility in shaping the future of African research. By adopting these strategies, they can move beyond a narrow focus on Western-centric research and actively contribute to building a vibrant research ecosystem that thrives within Africa. These strategies, implemented by funding agencies, can send a powerful message. They can demonstrate a commitment to supporting research that addresses African challenges and is disseminated through accessible channels that benefit African communities.

**Decolonizing research methodologies: embracing indigenous knowledge:** the current emphasis on Western research methodologies often overlooks the immense value of indigenous knowledge systems (IKS) in Africa [3-5]. Indigenous knowledge systems encompass the accumulated wisdom, practices, and beliefs of indigenous communities concerning their environment, health, and social life. Integrating IKS into research methodologies can lead to more holistic and culturally appropriate solutions to African challenges. Decolonizing research methodologies involves dismantling the dominance of Western epistemologies and embracing the richness of indigenous knowledge systems (IKS) present across Africa. Integrating IKS into research methodologies hold immense potential for: i) Enhancing research relevance: indigenous knowledge systems can provide valuable insights into local contexts, environmental conditions, and traditional approaches to health and well-being. This can lead to research that is more relevant to African communities and addresses their specific needs; ii) Promoting community ownership: incorporating IKS into research fosters a sense of ownership and collaboration among communities participating in research projects. This can lead to more sustainable research outcomes and increased trust between researchers and communities; iii) Unearthing new discoveries: indigenous knowledge systems can offer unique perspectives and methodologies that complement Western scientific approaches. This cross-pollination of knowledge can lead to ground-breaking discoveries and innovative solutions to challenges faced by Africa.

**Practically, decolonizing the research methodology may include but is not limited to some of the following interventions:** recognizing the value of indigenous knowledge systems: acknowledge the legitimacy and potential of IKS as a valuable source of knowledge for scientific inquiry. Research proposals that incorporate IKS methodologies alongside Western approaches should be encouraged. ii) Incorporating IKS into research design: researchers can engage with local communities to understand their traditional knowledge and practices relevant to the research topic. This could involve participatory research methods, where communities are actively involved in research design, data collection, and interpretation. iii) Collaborative research with indigenous communities: foster research partnerships with Indigenous communities, ensuring their active participation in all stages of the research process from problem identification to data collection, analysis, and dissemination of findings. This ensures that research respects traditional knowledge systems and benefits the communities involved. iv) Capacity building for indigenous knowledge systems integration: develop training programs and workshops for researchers on integrating indigenous knowledge systems into their research projects. This could involve training on traditional healing practices, ethnobiology, and methodologies for respectful and ethical collaboration with indigenous communities. v) Publication in African journals: encourage the publication of research that integrates IKS in reputable African journals. This not only disseminates valuable knowledge but also fosters its acceptance within the broader African scientific community. vi) Developing culturally sensitive methodologies: research methodologies need to be adapted to respect and value local cultural contexts. This might involve utilizing storytelling techniques, traditional healing practices, or indigenous resource management strategies within research frameworks. vii) Collaborative research with indigenous knowledge holders: partnerships with traditional healers, herbalists, and other knowledge holders within communities can be invaluable. Their expertise can be incorporated into research design, data collection, and interpretation, leading to more holistic and culturally relevant research outcomes. viii) Validating and integrating indigenous knowledge systems findings: research that integrates IKS needs to establish robust validation processes that ensure the credibility and reliability of findings. This could involve triangulation with other data sources, scientific testing of indigenous knowledge claims, and peer-review processes that acknowledge the value of IKS alongside Western scientific methodologies.

Decolonizing research methodologies is not about discarding Western scientific methods. It's about creating a more inclusive and holistic approach that recognizes the strengths of both traditional and Western knowledge systems. By embracing IKS, African research can gain deeper insights into local challenges, develop more culturally appropriate solutions, and ultimately empower communities to address their own priorities. This can lead to more effective and culturally appropriate solutions to complex challenges facing Africa. Promotion and tenure practices can be adapted to acknowledge the value of IKS by: i) Recognition of IKS expertise: promotion and tenure committees should acknowledge the expertise of researchers who collaborate with indigenous knowledge holders and integrate IKS methodologies into their research. This could involve recognizing co-authorship by indigenous knowledge holders or valuing research that effectively bridges the gap between Western science and IKS. ii) Training and capacity building: universities and research institutions can offer training programs that equip researchers with the skills necessary to understand and integrate IKS methodologies into their research. This could involve training in participatory research methods, ethnomedicine, and traditional ecological knowledge systems. iii) Development of ethical guidelines: rigorous ethical guidelines are crucial when incorporating IKS into research. These guidelines should ensure fair compensation for indigenous knowledge holders, intellectual property protection, and prior informed consent throughout the research process.

**The role of technology: empowering African researchers:** advancements in technology offer exciting possibilities for empowering African researchers and fostering a vibrant research ecosystem. Here are some key areas where technology can play a transformative role: i) Open access platforms: support and promote the development of robust open access platforms specifically for African journals. This ensures wider dissemination of research findings, increases accessibility for researchers and policymakers across the continent, and strengthens the visibility of African research globally. ii) Online collaboration tools: utilize online collaboration tools to facilitate research partnerships between African researchers dispersed across different countries. These tools can enable real-time communication, data sharing, and joint project development, fostering a more collaborative research environment. iii) Data sharing and management platforms: develop secure and user-friendly data sharing and management platforms specific to African research needs. This can facilitate data transparency, enable secondary data analysis, and foster collaboration among researchers. iv) Capacity building in digital research skills: equip researchers with the necessary skills to navigate the digital research landscape. This includes training on open access publishing platforms, online collaboration tools, data analysis software, and responsible use of social media for research dissemination. By harnessing the power of technology, we can bridge geographical divides, enhance collaboration, and create a more inclusive research environment for African scholars.

**Conclusion: collective action for transformative change:** the current P and T system in African academia is at a crossroads. We stand at a critical juncture, with the opportunity to rewrite the narrative and build a research ecosystem that empowers African scholars and fosters innovation relevant to the continent's needs. Dr. Abena's story represents the potential that is stifled by the current paradigm. Transforming P and T requires a multi-pronged approach. It necessitates a shift in perspective towards valuing African journals, recognizing diverse contributions of scholars, fostering collaboration, and decolonizing knowledge production. By incentivizing open access, redefining impact metrics, and nurturing a culture of intellectual risk-taking, we can empower the next generation of African researchers like Dr. Abena. We need a bold reimagining of P and T practices. Let's create a system that celebrates ground-breaking research, regardless of its publication origin. Let's build a research ecosystem that thrives on African ingenuity, addressing the continent's challenges head-on. Let us unlock the immense potential of African academia, ensuring that ground-breaking research like Dr. Abena's not only finds a platform but translates into tangible solutions that improve lives across Africa.

The duty to dismantle the current system that stifles African research and build a vibrant ecosystem that empowers African scholars, and African scholarship, foster ground-breaking research relevant to the continent, and positions Africa as a global leader in knowledge production is the collective responsibility of African researchers, African institutions, research partners, African policymakers, and global research funders. The potential for a transformed P and T system lies in its ability to ignite a new era of scientific discovery, empowering Dr. Abena and countless other brilliant African researchers to translate their ingenuity into solutions for a healthier, more prosperous Africa. Reimagining P and T practices in African academia requires a concerted effort from all stakeholders. Universities, research institutions, funding agencies, policymakers, and researchers themselves must all play a role in driving this transformative change.

Running a journal is a daunting task. Innovative ways of incentivizing journal editors and reviewers, most at times working on altruistic grounds, are required to maintain the quality, and timeliness of manuscripts accepted for publication. Indeed, this is one great path to fighting the proliferation of predator journals. Researchers: embrace innovative research methodologies, actively seek collaboration with indigenous communities, and publish their findings in reputable African journals. It is important to teach young researchers how to identify and shy away from predator journals. ii) Universities and research institutions: implement innovative P and T frameworks that value diverse research outputs, promote societal engagement, and create supportive environments for early-career researchers. A rethink of more meaningful metrics when it comes to academic promotions: mentorship, qualitative evaluations of publications (not limiting the quality of an academic to a number of papers published, or journal impact factors), innovation, participation in concrete knowledge translation initiatives (public talks, newspaper featuring, etc.). iii) Funding agencies: incentivize research addressing African challenges and published in African journals through dedicated funding streams, capacity-building programs, and revised evaluation criteria. iv)Policymakers: support the development of open access infrastructure, invest in digital research capacity building, and create a policy environment that encourages research relevant to African priorities. v) Society: publication of manuscripts in journals is unavoidable in the research and development enterprise. Citizen science approaches are needed to build public trust, raise awareness and the active participation of citizens in the science game, so they can hold policymakers accountable to increase and sustain investments in the research and publication spaces.
